# A3DyDB: exploring structural aggregation propensities in the yeast proteome

**DOI:** 10.1186/s12934-023-02182-3

**Published:** 2023-09-16

**Authors:** Javier Garcia-Pardo, Aleksandra E. Badaczewska-Dawid, Carlos Pintado-Grima, Valentín Iglesias, Aleksander Kuriata, Sebastian Kmiecik, Salvador Ventura

**Affiliations:** 1 Institut de Biotecnologia i de Biomedicina (IBB) and Departament de Bioquímica i Biologia Molecular, Universitat Autònoma de Barcelona, Bellaterra, Barcelona 08193 Spain; 2 Genome Informatics Facility, Office of Biotechnology, Iowa State University, Ames, IA 50011 USA; 3 Biological and Chemical Research Center, Faculty of Chemistry, University of Warsaw, Pasteura 1, Warsaw, 02-093 Poland

**Keywords:** Protein aggregation, Aggrescan 3D, AlphaFold, Yeast, *Saccharomyces cerevisiae*

## Abstract

**Background:**

The budding yeast *Saccharomyces cerevisiae* (*S. cerevisiae*) is a well-established model system for studying protein aggregation due to the conservation of essential cellular structures and pathways found across eukaryotes. However, limited structural knowledge of its proteome has prevented a deeper understanding of yeast functionalities, interactions, and aggregation.

**Results:**

In this study, we introduce the A3D yeast database (A3DyDB), which offers an extensive catalog of aggregation propensity predictions for the *S. cerevisiae* proteome. We used Aggrescan 3D (A3D) and the newly released protein models from AlphaFold2 (AF2) to compute the structure-based aggregation predictions for 6039 yeast proteins. The A3D algorithm exploits the information from 3D protein structures to calculate their intrinsic aggregation propensities. To facilitate simple and intuitive data analysis, A3DyDB provides a user-friendly interface for querying, browsing, and visualizing information on aggregation predictions from yeast protein structures. The A3DyDB also allows for the evaluation of the influence of natural or engineered mutations on protein stability and solubility. The A3DyDB is freely available at http://biocomp.chem.uw.edu.pl/A3D2/yeast.

**Conclusion:**

The A3DyDB addresses a gap in yeast resources by facilitating the exploration of correlations between structural aggregation propensity and diverse protein properties at the proteome level. We anticipate that this comprehensive database will become a standard tool in the modeling of protein aggregation and its implications in budding yeast.

## Background

The notable conservation of essential cellular structures and pathways such as cell cycle regulation, DNA repair, RNA processing, signal transduction pathways, metabolism, protein quality control mechanisms, or stress response, has positioned the budding yeast *Saccharomyces cerevisiae* (*S. cerevisiae*) as an ideal eukaryotic model organism. *S. cerevisiae* is also a reference expression system for the heterologous production of recombinant proteins. One of the major advantages of *S. cerevisiae* resides on its well-characterized and fully annotated genome. Indeed, several databases dedicated to yeast have been published, including the Saccharomyces Genome Database (SGD) [1], the Yeast Metabolome Database (YMDB) [2] or YEASTRACT+ [3]. Among them, the SGD database stands as the gold-standard repository with a wealth of integrated biological information for this microorganism. It also provides access to analytic tools to explore these data, enabling the discovery of functional relationships between sequence and gene products in fungi and higher organisms. In addition, the availability of comprehensive genomic resources, such as deletion libraries and collections of temperature-sensitive mutants, facilitates the identification and characterization of factors involved in different biological processes [4]. Indeed, *S. cerevisiae* stands out as one of the most widely employed model systems for studying protein aggregation in vivo. Notably, the yeast proteome is relatively small compared to higher eukaryotes, which makes it a privileged model for developing systematic studies and high-throughput screenings targeting protein aggregation [5]. Despite the relevance of this model organism in the field, aggregation propensity predictions are not currently reported in dedicated databases.

The identification of intermolecular interactions mediated by solvent-exposed aggregation-prone regions (APRs) embedded in the protein sequence has proven successful in predicting protein aggregation [6–10]. This approach is particularly suitable in the context of intrinsically disordered proteins (IDPs) or for newly synthesized polypeptide chains, but it often overpredicts when applied to folded proteins. Indeed, in folded proteins the identified APRs are often located within their hydrophobic cores or at inaccessible regions characterized by the presence of highly stable secondary structures [11, 12]. It is now widely accepted that globular proteins aggregate by the spatial clustering of often non-contiguous sequence regions of hydrophobic amino acids, forming structural APRs in the protein surface (STAPs) [13]. The aggregation may happen by local or global structural destabilization [14] or by stochastic fluctuations that lead to the exposure of previously buried APRs [15]. Consequently, considering a protein’s spatial environment becomes crucial in understanding the underlying forces driving its aggregation. In this context, we developed the Aggrescan 3D (A3D) algorithm [16–18], which makes use of the experimentally determined Aggrescan’s aggregation propensity scale [7, 19] and projects it into a three-dimensional protein structure. The versatility and accuracy of this algorithm have converted it into one of the default methods to study the aggregation of proteins in their natively folded states and how dynamic fluctuations and mutations impact this reaction.

Over the years, accurate predictions of structure-based aggregation propensities in yeast at the proteome level were hampered by the limited availability of structural information. However, the newly developed AlphaFold2 (AF2) database has released the prediction of thousands of structures from different organisms, including *S. cerevisiae* [20]. The overall quality of these computed models was shown to be comparable to experimentally determined structures [21]. Therefore, the newly reported structural information from AF2 allows the generation of proteome-wide repositories reporting yeast globular proteins’ aggregation properties.

Herein, we present the A3D yeast database (A3DyDB), which compiles the structure-based aggregation propensity predictions for 6039 *S. saccharomyces* protein models from the AF database [20]. Since many yeast proteins are not fully structured, the A3DyDB allows for the customization of jobs to adapt structural predictions according to AF2 confident cutoffs. The database also includes a tool for evaluating the influence of user-defined mutations on protein solubility and stability. We believe that the A3DyDB will serve as a useful resource for the study of protein aggregation in yeast. It will also allow the investigation of correlations between structural aggregation propensity and protein function, stability, architecture, location, and protein abundance, among other factors associated with protein aggregation. Ultimately, we illustrate the performance and utility of the database with selected case reports.

## Results

### A3DyDB summary and interface description

**Fig. 1:**
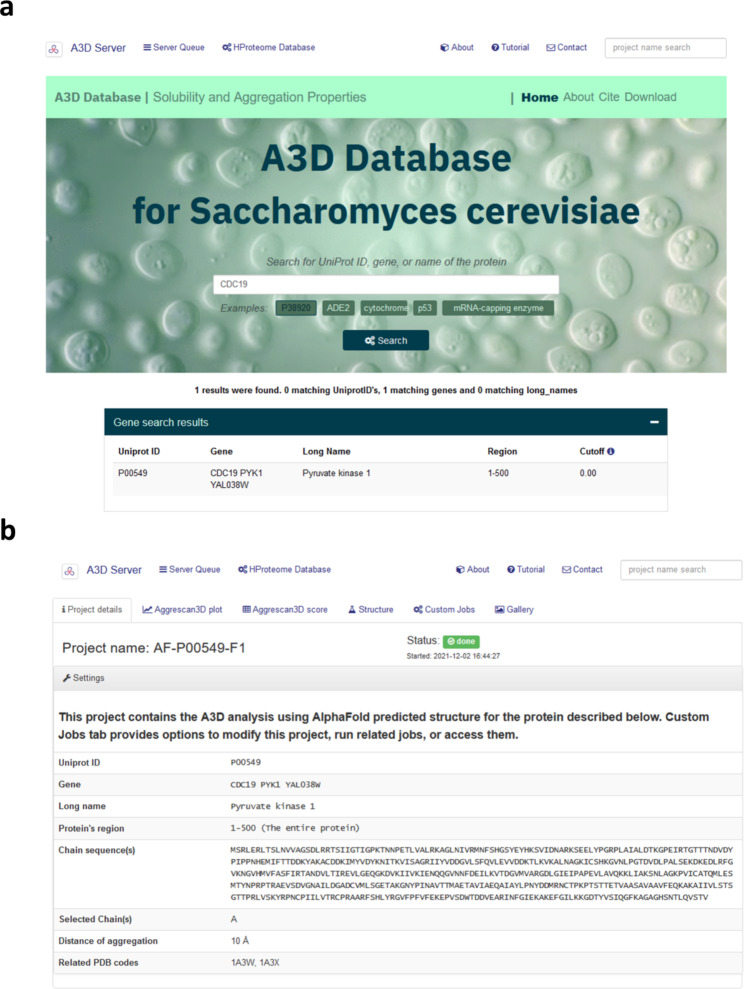
Overview of the A3DyDB home page and example of a search result for CDC19. (**a**) A3DyDB home page and query result for CDC19. (**b**) A3DyDB results page showing the project details tab for the CDC19 entry (Uniprot accession P00549)

**Fig. 2:**
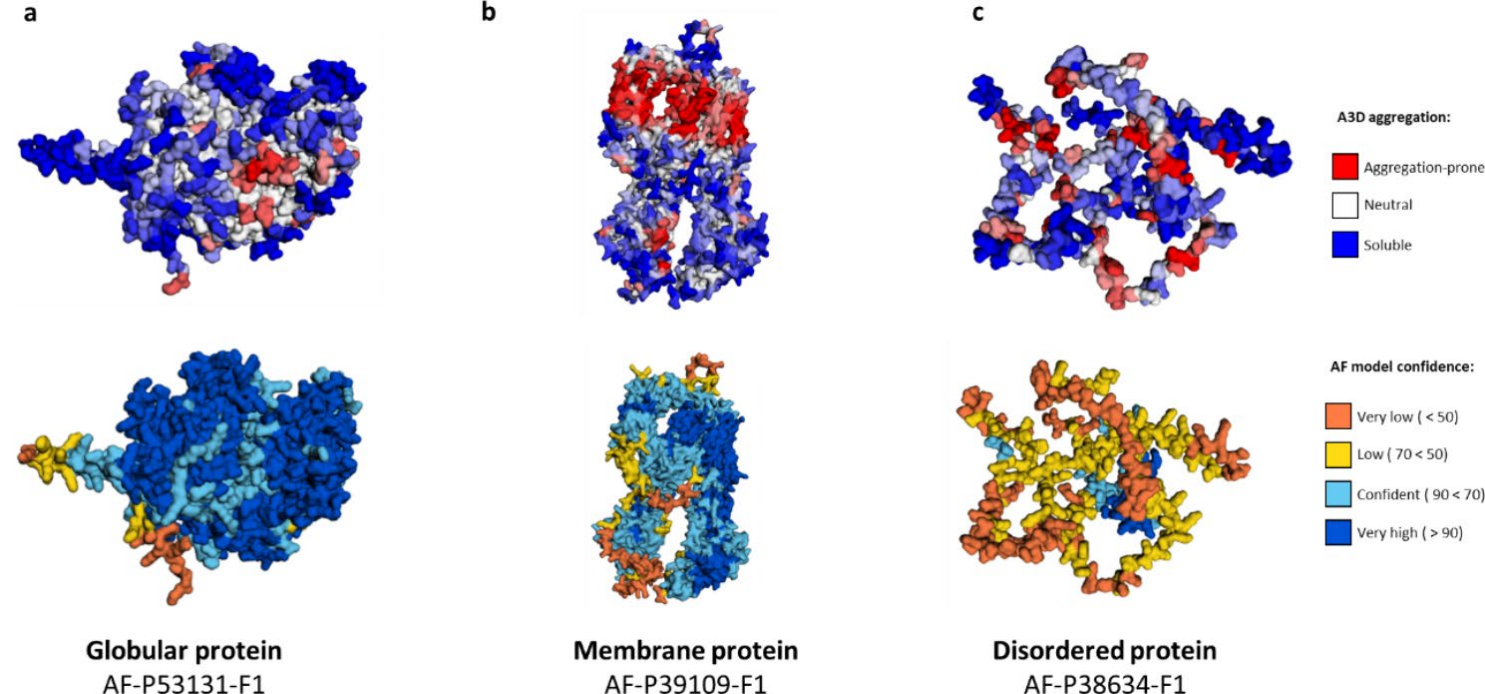
Examples of protein structural models as reported in the A3D Yeast database. Visualization of A3D predicted aggregation propensity ranges from blue (more solubilizing) to red (more aggregation-prone). A3D Yeast database provides the per-residue local confidence score (pDDLT), a metric that has been shown to inversely correlate with protein disorder [29]. Recent analysis suggests pDDLT scores < 70 usually correspond to unstructured in isolation. Examples of the A3D Yeast database output for (**a**) a globular protein, (**b**) a membrane protein and (**c**) a disordered protein are shown

The A3DyDB incorporates A3D predictions for 6039 proteins from the *S. cerevisiae* proteome. To perform aggregation predictions, we used a large dataset of structural models generated with AF2, which were downloaded from the AF database [20]. These structures were analyzed using the latest A3D implementation developed by our group [17, 22]. The resultant A3D data have been stored in the first comprehensive database describing the structure-based aggregation predictions for yeast (http://biocomp.chem.uw.edu.pl/A3D2/yeast). The A3DyDB is endowed with a search tab on the front page, which allows users to query for the content by using the gene, protein name, or Uniprot Accession (See Fig. 1a). Selecting entries from the query list leads to a page containing the A3D analysis. The analysis is distributed in different tabs containing the following information: (I) protein information and project details, (II) an interactive A3D score profile and annotation of transmembrane regions (only applicable to membrane proteins), (III) a detailed table containing the precalculated A3D scores and AF structure prediction confidence scores (pLDDTs), (IV) the protein structure colored by A3D and pLDDTs scores, (V) custom jobs with pre-calculated pLDDT cutoffs and (VI) collection of images. The A3DyDB *Project details* tab contains relevant information regarding the selected entry (Fig. 1b). *A3D plot/score* tabs display a detailed analysis of the per-residue aggregation propensity scores (A3D scores) and the profile and additional annotations for transmembrane and intermembrane proteins can be found in the subsequent sections. For each entry, two different structural models are provided in the *Structur*e tab (top panel in Fig. 2a). The top structure shows a per-residue prediction of the A3D aggregation propensity (A3D score), while at the lower structural representation AF2 confidence score (pLDDT) is depicted (lower panel in Fig. 2a).

Membrane proteins were also included in the database as A3D accurately predicts hydrophobic transmembrane segments as highly aggregation-prone regions (Fig. 2b). Although they do not contribute to typical aggregation mechanisms, these highly hydrophobic stretches are often inserted in membrane lipidic bilayers or involved in protein oligomerization. Based on Uniprot annotations, a total of 1219 transmembrane and intramembrane proteins from *S. cerevisiae* were identified. For these cases, we have included a complementary tab with relevant information, including predictions of consensus membrane segments from protein sequences by means of the TOPCONS server [23]. TOPCONS reports five different predictive algorithms, including OCTOPUS [24], Philius v [25], Polyphobius [26], SCAMPI [27], and SPOCTOPUS algorithms [28]. The inclusion of this module will facilitate the visual inspection and analysis of transmembrane regions, with the aim of performing comparative analyses.

A significant number of proteins from the A3DyDB contain structurally disordered regions. This is consistent with the observation that eukaryotes have a higher proportion of IDPs relative to bacteria or archaea [30]. AlphaFold2 outputs the pDDLT score, a per-residue estimate of the model’s confidence on a scale from 0 to 100 [20] which reports on the quality of the AF prediction [21]. Interestingly, regions of low confidence often correspond to intrinsically disordered regions (IDRs) [29]. Not surprisingly, manual data curation revealed that low pLDDT scores might result in misleading A3D predictions, either because these solvent-exposed regions are more exposed or compact in the model than they should be. A significant number of yeast proteins showed the presence of regions with low predicted accuracy (low pLDDT score in Fig. 2c). After testing different pLDDT thresholds, we decided to precompute A3D on top of three different AF models for each protein entry: the full-length protein model and two additional models in which residues with pLDDT < 70 or residues with pLDDT < 50 were removed (see **Figure S1**). These precalculated models can be directly accessed from the *Custom Jobs* tab. The A3DyDB implementation also allows users to model the effects of custom mutations on the stability and aggregation propensity of a given particular protein entry using the FoldX force field [31]. Using the mutation editor at the *Custom Jobs* tab it is possible to evaluate the effects of single or multiple mutations in a custom A3D analysis. These new jobs will be immediately listed and accessible in the A3DyDB queue.

## Discussion

Protein aggregation is increasingly recognized as a contributing factor to various pathologies in eukaryotes [32] and constitutes a major limitation to produce functional recombinant proteins in yeast [33]. Numerous past studies, mostly performed using simple prokaryotic and eukaryotic model organisms such as bacteria and yeast, have led to a detailed understanding of how highly aggregation-prone proteins form insoluble species and how these proteins are toxic for the cells [34]. These seminal investigations have allowed the identification of important principles of protein aggregation, which has led during the last decade to the development of a series of predictive algorithms to identify aggregation-prone sites [35]. Our current understanding of the structural landscape of the yeast proteome has radically changed with the development of deep-learning-based approaches, such as RoseTTA fold [36] or AF2 [21]. This new wealth of structural data can be exploited to predict the aggregation properties of the whole yeast proteome and undertake the redesign of yeast proteins to improve their solubility and stability for diverse purposes. Herein, we have launched the A3DyDB, which contains the precomputed aggregation predictions for the *S. cerevisiae* proteome, and we have tested the performance of the database in a variety of case reports. Below, we provide selected case examples demonstrating the suitability of our comprehensive repository in diverse scenarios.

### Case examples

#### Exploiting the A3DyDB to study cellular organization and metabolism in yeast

Yeast organisms live in a wide range of different environments which require local adaptations to transient conditions [37]. For this reason, proteins have evolved to self-organize in the cellular milieu in response to specific stimuli such as nutrient starvation. Under these circumstances, metabolic enzymes from yeast proteins undergo a widespread reorganization into reversible punctate cytoplasmic foci that are disassembled when the stress is released [38]. Interestingly, the evidence strongly suggests that the formation of reversible protein assemblies, specific to metabolites, is potentially widespread in the realm of cell biology [38]. In this context, the ability of proteins to form assemblies is closely linked to their propensity for aggregation. Linear aggregation predictors such as TANGO [39] have been used to study differences in protein aggregation in these dynamic assemblies [38], but most of the proteins involved in foci formation contained globular regions which require dedicated structure-based aggregation predictive tools.

**Fig. 3:**
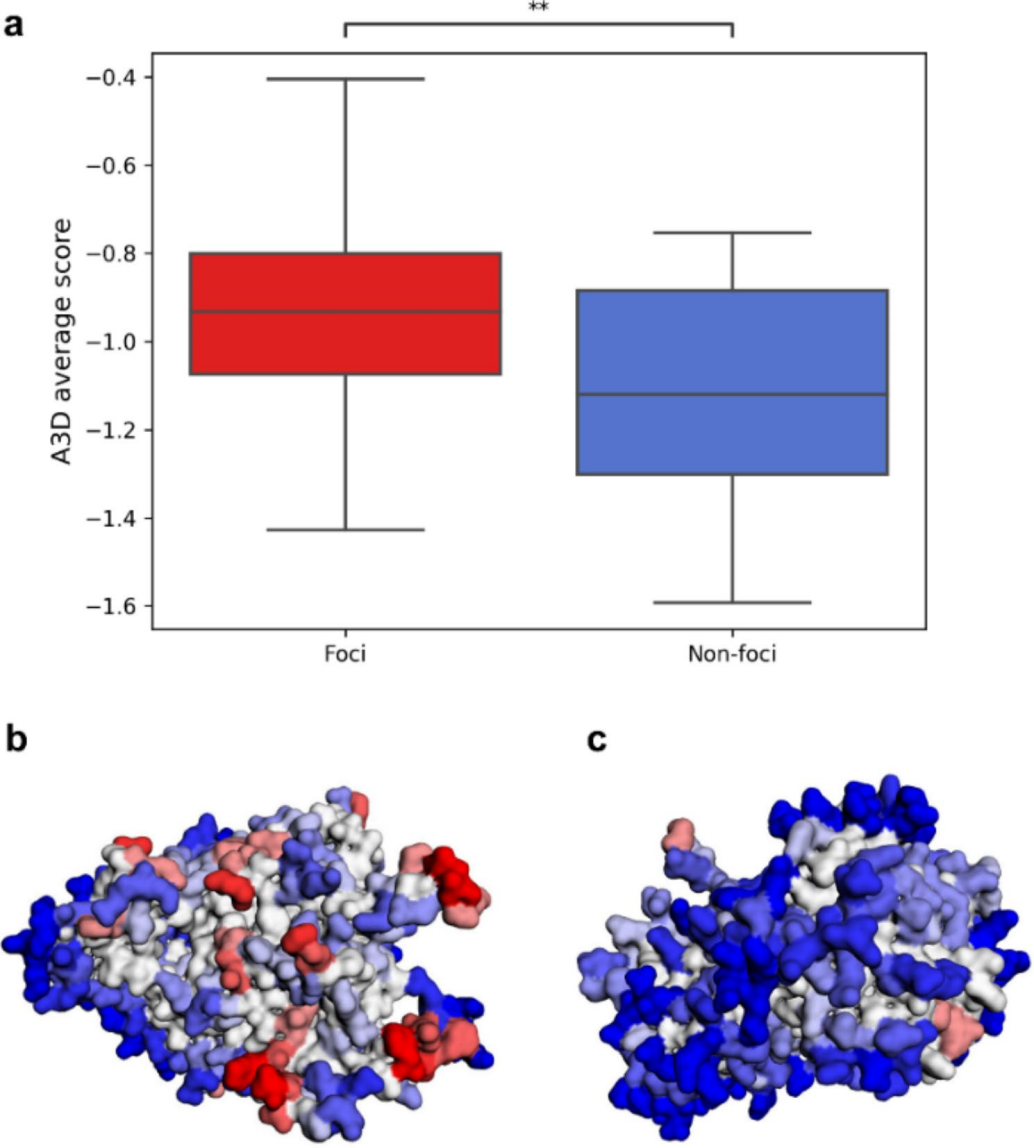
Structural aggregation propensity differences between foci and non-foci forming proteins from yeast. (**a**) The A3D average score is higher for proteins observed to form punctate foci (in red) than those that did not form (in blue). A statistically significant difference between the two groups was observed (Mann-Whitney-Wilcoxon two-sided test*-t*; p = 0.0047). The A3D predictions from AF ARO2 (AF-P28777-F1) (**b**) and TIF2 (AF-P10081-F1) (**c**) structures, two representative cases of foci and non-foci proteins respectively. The foci-forming protein ARO2 presents a larger number of STAPs than the non-foci protein TIF2.

Herein, we used the data from the A3DyDB to investigate possible differences in structural aggregation between the 180 foci and 27 non-foci forming proteins described by Narayanaswamy et al. [38] (Supplementary Table 1). Our results showed that foci-forming proteins had a significantly higher A3D average score than non-foci-forming proteins (Fig. 3a). Indeed, the visual inspection of proteins from the two independent datasets revealed that foci proteins, such as ARO2, contain a larger number of STAPs than non-foci proteins like TIF2 (Fig. 3b and c, respectively). Overall, it seems that protein organization in yeast is a very dynamic process that could be, at least, partially understood by protein aggregation. In this framework, the release of thousands of structural predictions in the yeast A3D database can help in identifying STAPs in several proteins involved in cellular reorganizations.

#### Predicting STAPs to study functional protein assemblies

**Fig. 4:**
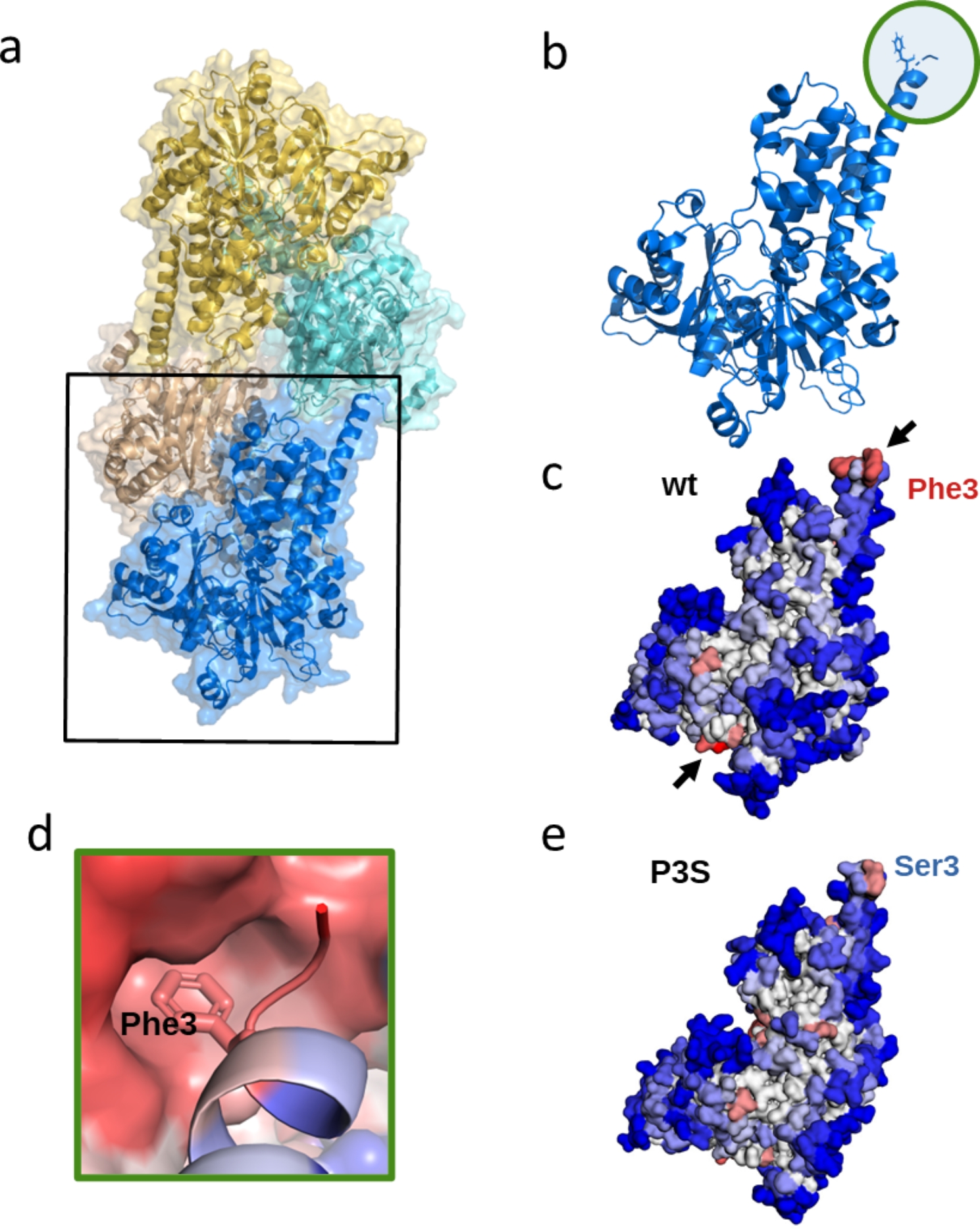
A3D predictions for yeast hexokinase GLK1 self-assembly. (**a**) Cryo-EM structure of the yeast hexokinase GLK1 filament (PDB: 6PDT). (**b**) Structure of a GLK1 monomer showing the location of the N-terminal Phenylalanine residue involved in filament formation (Phe3, indicated with a circle). (**c**) A3D prediction of the wild-type active monomeric GLK1 obtained from the A3DyDB. Predicted aggregation propensities are colored in the structure ranging from blue (more solubilizing) to red (more aggregation-prone). The position of the two predicted hydrophobic STAPs is indicated with arrows. (**d**) Detailed representation of the insertion site of Phe3 into the C-terminus hydrophobic cavity. (**e**) A3D prediction of the Phe3Ser (P3S) solubilizing mutant. This solubilizing mutation eliminates filament polymerization

We have investigated a case example in which STAPs are important for mediating functional protein-protein interactions. The actin fold is found in cytoskeletal polymers, chaperones, and various yeast enzymes involved in metabolic pathways. Most actin-fold proteins, such as the carbohydrate kinases are monomeric proteins and do not polymerize. However, it has been recently reported that the *S. cerevisiae* glucokinase GLK1 can form polymers in response to its substrates and products (Fig. 4a) [40]. The polymerization of this actin-fold protein inhibits its kinase catalytic activity, a mechanism directly coupled to cell viability and the adaptation of the yeast to stochastic changes in the environment. Recently, cryo-EM studies have revealed that glucokinase GLK1 *from S. cerevisiae* is able to form two-stranded filaments with a molecular architecture different from that of cytoskeletal polymers [40]. These filaments are built up by GLK1 monomer stabilized by hydrophobic interactions between GLK1 subunits along a strand. In this structure, a solvent exposed Phe3 of one GLK1 subunit is inserted into the hydrophobic pocket at the C-terminus of the next GLK1 moiety, effectively mediating the stabilization of the filament (Fig. 4b).

A detailed analysis of the structural aggregation propensity of the monomeric GLK1 was obtained from the AF2-derived model collected in A3DyDB (Fig. 4c). Our analysis showed the presence of two strong STAPs in this protein that overlap with the observed GLK1 oligomerization interfaces, where the N-terminus Phe3 is inserted in the hydrophobic C-terminus cavity (Fig. 4d). This is consistent with the view that functional and aberrant polymerization surfaces share very similar physicochemical properties and do frequently overlap [13, 41]. Garner and coworkers mutated the N-terminal Phe3 involved in GLK1 assembly contacts to Ser, to change the non-polar character of this [21] protein position [40]. Based on our A3D predictions, this mutation significantly decreases the potency of the N-terminal STAP (Fig. 4e), which coincides with the experimental observation that it eliminated polymerization both in vitro and in vivo.

#### Using the A3DyDB to study membrane proteins

**Fig. 5:**
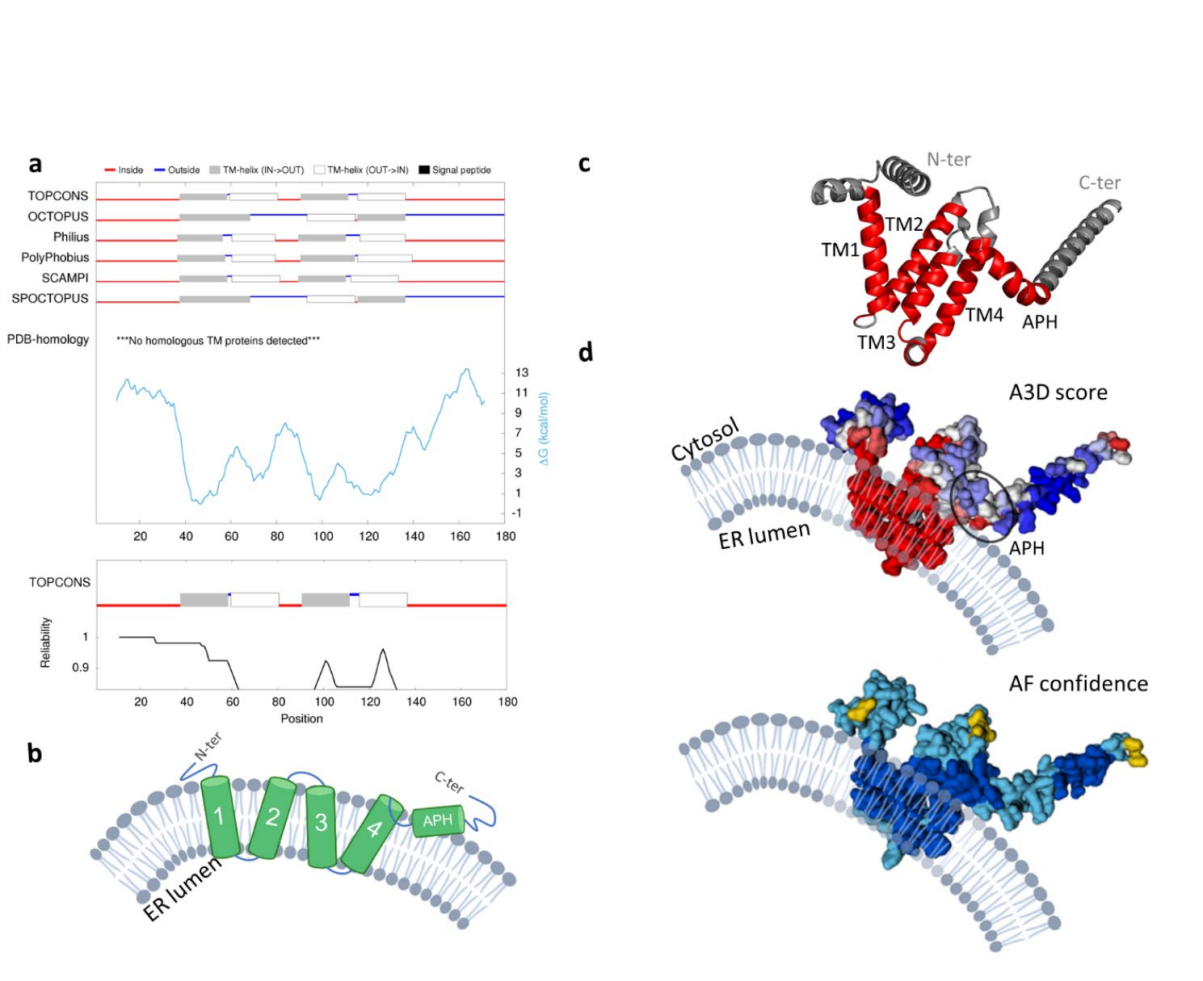
A3DyDB allows the study of membrane proteins and their functional implications. (**a**) A3DyDB results page or Transmembrane regions tab for YOP1 entry (Uniprot Accession Q12402). (**b**) Scheme of YOP1 predicted topology deduced from A2DyDB TOPCONS predictions and previous experimental data [44, 46]. (**c**) YOP1 AF2 model (AF-Q12402-F1), showing the location of predicted TMs and APH regions. (**d**) A3D analysis (upper panel) and AlphaFold2 (AF2) model confidence score (lower panel). Note that YOP1 exposes STAPs to the hydrophobic interior of the membrane bilayer and at the APH region

The endoplasmic reticulum (ER) network is built up by tubules with high membrane curvature in cross-section, which are generated and stabilized by reticulons and receptor expression-enhancing proteins (REEPs). Reticulons and REEPs are integral membrane proteins resident at the ER that are evolutionary conserved across all eukaryotes [42, 43]. These proteins share a common architecture that has been also identified in other human proteins that function as ER-phagy intramembrane receptors (i.e. ATG40 in *S. cerevisiae* and FAM134B in mammals) [44, 45]. Here, we used the A3DyDB to investigate the structure and aggregation propensities of YOP1, as a model membrane protein (Fig. 5).

YOP1 is a yeast reticulon that is highly enriched in the tubular portions of the ER and virtually excluded from other regions, which induces high membrane curvature of tubules by an unknown mechanism. Indeed, deletion of the reticulons and YOP1 in yeast has been linked to the loss of tubular ER. Previous studies have suggested that YOP1 generates high membrane curvature by hydrophobic insertion and scaffolding mechanisms, which is mediated by the insertion of the TM regions on the highly hydrophobic lipid bilayers [47–49]. The suggested mechanism relies on functional data and the protein’s structure, but its experimental verification remains incomplete at this stage.

We have analyzed the presence of membrane segments at the transmembrane regions tab from the A3DyDB output. We found that YOP1 has two pairs of trans-membrane (TM) segments, which is one of the characteristic features of reticulons (Fig. 5a). These TM regions are well predicted by the different algorithms implemented in the database and by the consensus TOPCONS prediction, which is accompanied by an overall high prediction reliability (Fig. 5a). Next, we have compared the structure-based aggregation predictions from A3D with the proposed membrane topology. Most of the TM regions were predicted as large highly aggregation-prone segments, as expected for such hydrophobic regions. The main four STAPs in YOP1 were followed by an additional strong short aggregation-prone stretch (residues Ile143-Ile152), a region that matches with an amphipathic helix (APH) that is required for maintaining the characteristic reticulon’s ER-tubule localization (Fig. 5c and d). Both the TMs and APH have been shown as essential elements to generate high membrane curvature and for maintaining relevant protein-protein interactions [46]. It has been previously demonstrated that YOP1 undergoes homotypic and heterotypic oligomerization [46, 47, 50, 51]. This behavior was mostly due to homotypic interactions mostly between the TMs regions, as suggested by Cystein-based crosslinking experiments [50, 51]. Taken together, our results indicate that STAPs are present in YOP1, and it is likely that these regions are involved in membrane binding, as well as in maintaining YOP1 oligomerization interaction interfaces.

## Conclusions

The A3DyDB provides a unique repository of structural aggregation predictions for thousands of yeast structures collected in the AF database, a resource that for a long time remained elusive due to the limited amount of available structural data. Given the importance of *S. cerevisiae* as a model organism in aggregation, we see A3DyDB as a valuable resource to inspect STAPs in yeast proteins and find associations between aggregation propensity and other functional aspects of yeast biology. Besides, A3DyDB can be used to analyze the effect of mutations on the 3D surface of proteins and engineer variants that could become more stable and soluble. The presented here *in silico* approach can serve to make faster and more cost-efficient yeast mutants for different applications such as reconstructing metabolic networks, improving the solubility of endogenous proteins, recombinant protein production, and anticipating improved protein variants in synthetic biology approaches.

## Methods

### Data collection and A3D analysis

*S. cerevisiae* (UP000002311) protein structures (n = 6039) were downloaded from the AF database (October 20, 2022; structural model version v4, available at https://ftp.ebi.ac.uk/pub/databases/alphafold/v4/UP000002311_559292_YEAST_v4.tar) and run with A3D in static mode with a distance of aggregation analysis of 10Å and FoldX-based energy minimization for stability calculations. Custom jobs were created for all predicted structures with two defined AF cutoffs 50 and 70, with residues of pLDDT < = 50 or < = 70 removed for the A3D aggregation prediction.

### Database construction

The user interface of the A3DyDB online database was developed utilizing HTML and integrated with custom JavaScript functions to enhance interactivity. The visual design of the website is a combination of standard Bootstrap components along with a touch of custom CSS styles. An Apache2 web server is employed to host the website, leveraging MySQL integration to handle data storage, retrieval, and querying of the pre-calculated A3DyDB entries. Interactive plots are dynamically generated using the D3.js library, while molecular visualization tasks are handled by the Open Source PyMOL tool [52]. Additionally, the database is seamlessly integrated with the A3D Server, enabling direct submission of custom mutation analysis. The transmembrane analysis for membrane proteins was performed with the consensus algorithm TOPCONS [23], which includes predictions from OCTOPUS [24], Philius [25], Polyphobius [26], SCAMPI [27], and SPOCTOPUS [28].

### Foci and mutation structural analyses

Foci (n = 180) and non-foci (n = 27) forming proteins were obtained from [38]. Structural aggregation propensities for both protein datasets were obtained from the yeast A3DyDB. Statistical significance between variables and/or datasets was assessed with Mann-Whitney-Wilcoxon two-sided test with Bonferroni correction. p-value was marked with asterisks to better convey statistical significance (p > 0.05 (ns), p ≤ 0.05 (*), p ≤ 0.01 (**), p ≤ 0.001 (***), p ≤ 0.0001 (****)).

GLK1 mutation analysis was performed with A3DyDB mutation mode under the Custom jobs tab. Structural representations of A3D mutants were obtained from the Structure tab visualization tool.

## Data Availability

All data generated or analyzed during this study are included in this published article and the A3DyDB is freely available at http://biocomp.chem.uw.edu.pl/A3D2/yeast.
